# Annotate-it: a Swiss-knife approach to annotation, analysis and interpretation of single nucleotide variation in human disease

**DOI:** 10.1186/gm374

**Published:** 2012-09-26

**Authors:** Alejandro Sifrim, Jeroen KJ Van Houdt, Leon-Charles Tranchevent, Beata Nowakowska, Ryo Sakai, Georgios A Pavlopoulos, Koen Devriendt, Joris R Vermeesch, Yves Moreau, Jan Aerts

**Affiliations:** 1 KU Leuven, Department of Electrical Engineering-ESAT, SCD-SISTA, Kasteelpark Arenberg 10, B-3001, Leuven, Belgium; 2 IBBT Future Health Department, Kasteelpark Arenberg 10, B-3001, Leuven, Belgium; 3 KU Leuven, Centre for Human Genetics, University Hospital Gasthuisberg, Herestraat 49, 3000 Leuven, Belgium

## Abstract

The increasing size and complexity of exome/genome sequencing data requires new tools for clinical geneticists to discover disease-causing variants. Bottlenecks in identifying the causative variation include poor cross-sample querying, constantly changing functional annotation and not considering existing knowledge concerning the phenotype. We describe a methodology that facilitates exploration of patient sequencing data towards identification of causal variants under different genetic hypotheses. Annotate-it facilitates handling, analysis and interpretation of high-throughput single nucleotide variant data. We demonstrate our strategy using three case studies. Annotate-it is freely available and test data are accessible to all users at http://www.annotate-it.org.

## Background

### Context

With the advent of massively parallel high-throughput sequencing technologies and the increasing availability of reference genomes, new opportunities emerge for discovering genome-wide variation across individuals and populations, at both the large-scale level (deletions, duplications, and rearrangements) and the base-pair level (single nucleotide variants and small indels and repeats). As more and more sequence data are produced, accurate assessment of the frequency of variants in specific subpopulations (patients versus controls or any phenotypically different populations) is vital to the interpretation of how these variants segregate across populations. International projects, such as the 1000 Genomes Project [1] and the Hapmap Project [2], have been set up to assess the genetic variation across large groups of 'normal' human individuals. Full-genome [3], whole-exome [4-6] or targeted gene panel [7,8] sequencing studies of individuals struck by Mendelian disorders can aid in identifying the genetic cause for these diseases; for example, by leveraging publicly available data, under the assumption that these rare variants do not occur in the normal population. Furthermore, trio sequencing of an affected child and of his or her parents can identify *de novo *variants in sporadic cases of genetic disease [9,10].

Because of the large size and complexity of next-generation sequencing data sets, new computational and statistical methods for analyzing and interpreting the data are required to accurately find the variation of biological interest. To transform raw sequencing data into variation data, we need to undertake the following steps: (1) sequence alignment, (2) variant calling, (3) variant annotation, and (4) variant interpretation [11,12]. In this study, we mainly focus on the latter two steps. After sequence alignment and variant calling, we end up with a list of variants with their genomic coordinates and the variant alleles that differ from the reference sequence. Based on current knowledge of functional elements annotated in the human genome sequence, overlapping variants within the annotated features are found and the impact on RNA and protein sequence level is computed. Variant lists can be reduced further by applying functional impact prediction tools, such as Polyphen2 [13], SIFT [14], FoldX [15], and others [16]. Such tools are computationally intensive and require dedicated computing infrastructure to run variants in a high-throughput automated fashion. To tackle this problem, precomputed whole-genome predictions for popular tools are available in Ensembl and in dedicated databases [17]. Aggregated functional impact scores, such as CAROL [18], are also useful as these usually perform better in classification benchmarks for disease-causing mutations but are often not provided by current annotation tools.

These annotated variants can then further be interpreted by filtering based on either quality, functional, or genetic criteria. These filtered annotated data are then interpreted by either collapsing the data into the desired functional units, often genes, or performing formal statistical association analysis methods [19].

Several software tools have already been developed to tackle the aforementioned analysis steps. Some of these tools, such as ANNOVAR [20], TREAT [21], VarioWatch [22], SeqAnt [23], and Anntools [24], have been specifically designed to handle the annotation task and use diverse data sources in order to achieve this (Table 1). Other tools focus solely on the interpretation step and facilitate tasks such as easy filtering of data (VarSifter [25]), ranking based on putative pathogenicity (Var-MD [26]) or do more complex types of analysis by performing association analyses or looking at different underlying genetic disease models (VAAST [27]). A variety of tools try to be more comprehensive and provide a streamlined approach by tackling both steps (KGGSeq [28], SVA [29]).

**Table 1:** Comparison of features of different tools for next-generation sequencing annotation and interpretation

	ANNOVAR	TREAT	VAAST	VarSifter	Var-MD	KGGSeq	SVA	Anntools	VarioWatch	SeqAnt	Annotate-it
Interface	Command line	Command line	Command line	Graphical	Python script	Command line	Graphical	Command line	Web	Web	Web
Indels	x	x	x	x		x	x	x		x	
SNPs	x	x	x	x	x	x	x	x	x	x	x
miRNA annotation	x							x			x
Regulatory region annotation	x	x				x		x			
Custom annotation	x						x	x			
Gene annotation	x	x				x	x	x	x	x	x
Conservation scores	x	x				x				x	x
LRT	x					x					x
MutationTaster	x					x					x
Polyphen2	x	x				x					x
SIFT	x	x				x			x		x
Aggregate scores						x					x
Haploinsufficiency prediction											x
OMIM		x					x		x		x
NHLBI Exomes	x										x
200 Danish Exomes											x
1000 Genomes	x	x				x	x	x	x	x	x
dbSNP	x	x				x	x	x	x	x	x
Gene prioritization											x
Literature retrieval						x			x		x
Gene ontology							x		x		x
PPI/complexes						x					x
Pathway information		x				x	x		x		x
Gene expression		x									x
Statistical analysis			x				x				x
Filter	x	x		x	x	x	x				x
Complex filtering rules				x		x	x				x
Cross-sample querying				x	x	x	x				x
Genetic inheritance models			x	x	x	x	x			x	x
Data sharing											x
Visualizations		x					x		x	x	x

OMIM, Online Mendelian Inheritance in Man; PPI, protein-protein interaction.

Although current tools have matured significantly, they still possess several limitations. First there is the 'red queen' annotation problem. As functional information is constantly evolving, end users are forced to keep data sources and variant annotation up to date with every release of new information. Although some annotation tools make it easy to update annotation sources, this can still be a problem when trying to use previously acquired data sets in a related setting without repeating the complete annotation process. Secondly, interpretation is often done at the level of the individual sample, whereas cross-sample analysis is often left to the end user. This requires computational expertise when dealing with large data sets. Additionally different biological contexts might require a different combination of analysis techniques (that is, case/control studies versus familial or trio studies). Also, these analysis techniques are often phenotype-naive and disregard existing *a priori *biological knowledge of the particular disease or phenotype under study. Thirdly, when working in a collaborative setting, keeping track of individual files, annotation versions, and variants can prove difficult and easy sharing of variation, validation, sample, and experiment metadata is usually crucial. This is especially the case in the analysis of Mendelian disorders where cases are often spread globally across research institutions.

### Aim

To tackle these hurdles, we developed a framework, called Annotate-it, that provides experimentalists with a Swiss-knife approach for the interpretation of single-nucleotide variants, providing features such as automated annotation, prioritization of mutated genes, cross-sample querying and data management. We expand on existing comprehensive annotation and interpretation frameworks by integrating more annotation sources and different established state-of-the-art analysis techniques. We also integrate two different gene prioritization techniques: AGeneApart [30] and Endeavour [31]. AGeneApart mines MEDLINE abstracts to discover known genes or potentially new genes linked to the phenotype whereas Endeavour ranks genes based on similarity profiling compared to known disease genes. The latter method is more suitable for finding novel disease genes as it does not require a direct relationship between gene and disease [32]. These methods allow us to rank mutated genes taking the phenotype into account. Finally, because of its web-based interface, we allow for a collaborative workflow and the management of shared or public data, as well as a wide range of visualizations.

## Implementation

### Annotation and filtering methodology

Multiple annotation sources are supported, which can be classified into gene level and variant level information. Genes are annotated using Gene Ontology [33] terms, Online Mendelian Inheritance in Man (OMIM) and haploinsufficiency predictions [34]. We also provide pathway information from KEGG [35], BIOCARTA, and Reactome [36], as well as protein-protein interaction and protein complex data from STRING [37] and the Corum project [38]. Additionally, we provide statistical phenotypic associations with known chromosomal aberrations and phenotypic ontologies based on text-mining of recent MEDLINE releases [30]. Tissue-based expression information from the eGenetics/SANBI data set is also included to search for genes that are expressed in particular tissues of interest.

Information on the individual variants include PhastCons and Phylop scores (for primate, placental mammal and vertebrate multiple alignments) and presence in dbSNP, 1000 Genomes Project, and the 200 publicly available Danish exomes [39]. Also functional impact prediction scores from SIFT [14], Polyphen2 [13], LRT [40], and MutationTaster [41] are extracted from dbNSFP [17]. An aggregate score of SIFT and Polyphen2 is also computed with CAROL [18], which has been demonstrated to perform better than individual scores at predicting deleteriousness of nonsynonymous variants.

Annotate-it has a flexible query system that allows answering complex cross-sample questions in an easy and quick way without any need for computational expertise. Using this interface, users can quickly check different inheritance hypotheses (for example, recessive or dominant models, or *de novo *occurrence of variants in parents-child trio analysis) depending on the available familial information. It also permits filtering based on consequence type, minimum and maximum coverage, minimum and maximum variant allele frequency, heterozygosity call, presence in dbSNP, 1000 Genomes Project, the NHLBI exome variant server and 200 Danish exomes, variants present in other samples, and conservation scores (Additional file 1). Annotate-it also allows screening a user-defined (or automatically generated) panel of genes of interest. Ultimately, these filtering steps will result in a list of genes (or exons) ordered by user-specified criteria (for example, present in given number of samples, number of unique alleles) (Additional file 2). Genes can then be further inspected by consulting all the available information that Annotate-it aggregates. This information contains associated phenotypes, automated literature retrieval, links to OMIM, region-based conservation plots, STRING or protein complex interaction partners and gene ontology terms (Additional file 3).

### Interpretation and statistical analysis

In addition to the widely used collapsing method of disease-gene discovery, where variants across affected samples are collapsed at the gene level and counted, the user can also perform formal statistical analysis using the methodology proposed by Ionita-Laza for case/control studies [19]. This type of statistical methods has the added benefit of computing an approximate *P*-value for individual genes and of taking into account inherent background variation in genes due to factors such as gene size or hypermutable regions. However, they do require a significant amount of control samples in order to reach satisfying statistical power (n > 50). The analysis can be applied directly on the imported data without any reformatting or performing any command line actions.

The user can also provide a particular phenotype using Human Phenotype Ontology terms that can be used to derive genome-wide rankings produced by AGeneApart and Endeavour. These rankings are precomputed for all Human Phenotype Ontology terms for which at least five gene-phenotype associations are known, which can be used as a training set for the prioritization algorithm.

Furthermore the user can investigate mutation patterns of interaction partners of genes of interest. This is achieved by integrating the STRING and CORUM protein-protein interaction networks. This can be of particular interest when the cause of disease is expected to be oligogenic, such as diseases with some degree of phenotypic variability.

### Metadata and collaboration

Additional metadata can be linked to samples and experiments. Samples are entities within experiments and can contain phenotypic information from different integrated ontologies (for example, European Paediatric Cardiac Code [42], London Dysmorphology Database [43], Human Phenotype Ontology [44]), gender, the date of sequencing, batch number, and other data. Metadata allow organizing and querying samples across multiple experiments. The experiments themselves can also store metadata, such as the software and parameters used for alignment and variant calling. Within an experiment, variants can be marked for validation, and validation information can be stored and reviewed. Promising candidate genes can be organized into gene lists when dealing with many collaborators or complex study setups. Gene lists can also be used as masking filters to either study gene specific gene panels or exclude sets of genes that are not of interest. An experiment-wide permission system with different roles (owner, editor, user) allows collaboration amongst different researchers and also allows the public sharing of data.

### Visual analytics

**Figure 1:**
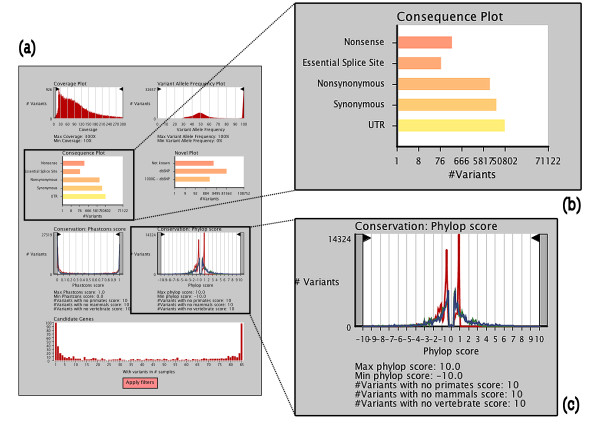
**Screenshot of interactive graphical filter**. **(a) **In Annotate-it, the graphical interactive filter allows the user to view the distributions of a number of parameters and interactively set filter settings. Filters can be adapted by dragging thresholds or clicking to select/deselect certain types of variants. Filtering on one parameter shows the impact on all other parameters in near real time. Plots included at the time of writing are (clockwise starting from upper left) coverage, variant allele frequency, presence in public databases, Phylop scores, the number of candidate genes hit in a given amount of samples, and Phastcons scores. Once satisfied the user can apply the filter settings and inspect the resulting gene lists. **(b, c) **Zoomed in portions of the interactive filter show a bar plot showing the proportions of variants of different consequence types (b) and the distribution of conservation scores for (red) primates, (blue) mammals, (green) vertebrates (c).

Visual analytics is gaining ground for data exploration in the context of big data. The integrated interactive graphical filter (Figure 1) visualizes the different distributions of variants (for example, coverage, consequence types, conservation scores, variant allele frequency) and shows the impact of different filtering thresholds in real time, allowing selection of optimal thresholds. This is particularly useful in an exploratory phase to get a grasp on the characteristics of the data set and on how filter settings will affect these characteristics. Once optimal thresholds have been selected, they are easily applied in a subsequent discovery phase where the main goal is to select candidate genes of interest.

Calculating statistics for such large data sets in real-time is computationally taxing; therefore, Annotate-it's interactive filter can currently only be run on a subset of the variants (maximum 200,000 variants). Further research in the development of advanced data structures and browser infrastructure is needed to cope with the running time complexity of these approaches. As user interaction and feedback increases, we aim to optimize the informative value of such interconnected graphs and to improve the throughput of these visualization techniques.

### Technical specifications

Annotate-it is programmed in the Ruby programming language and makes use of the Ruby on Rails framework [45] for its web interface. Currently, Annotate-it supports both hg18 (NCBI36) and hg19 (NCBI37) genomic builds. The annotation process leverages existing libraries, such as the Ruby Ensembl-API [46] and BioRuby [47], to enable programmatic access to locally run mirrors of the latest Ensembl [48] versions for any given genomic build. MySQL [49] was chosen to store variant information in a database. Nevertheless, Annotate-it's architecture is completely technology independent and therefore switching to different Rails-supported databases requires little effort. For large data sources containing billions of data points (such as base-wise conservation scores), we rely on Tokyo Cabinet [50] key-value stores for quick access. Interactive visualizations were implemented using Processing [33] and integrated into the web-interface as java applets.

Computationally intensive tasks (for example, complex predictions based on protein structure analysis) can be accomplished in a distributed manner by installing the Annotate-it client and the software one wishes to run on any computing node that has direct network access to the Annotate-it server. In this way, computing power can be scaled up on demand, either locally or by the use of cloud-based systems. Variant files can be uploaded and imported in the correct data structures at a pace of 500 variants per second using the current computing infrastructure. Previously unseen, and thus yet unannotated, variants are processed at a rate of approximately 85 variants per second. Computing infrastructure can be easily expanded to face increasing demand using elastic cloud technologies. This process is run completely at the server-side and requires no user interaction.

Input files of different formats are supported. So far, support includes Varscan [51], Atlas-SNP2 [52], Samtools [53] variant calling format, Genome Analysis Toolkit's VCF4 [54], Roche 454 GSMapper, and Illumina CASAVA output. Multiple files of different formats or sequencing technologies can be transparently combined for a single sample. Gene and variant lists can be exported to tab-separated value files based on the used filter-settings if needed.

### Generation of Miller syndrome semi-synthetic data sets

**Figure 2:**
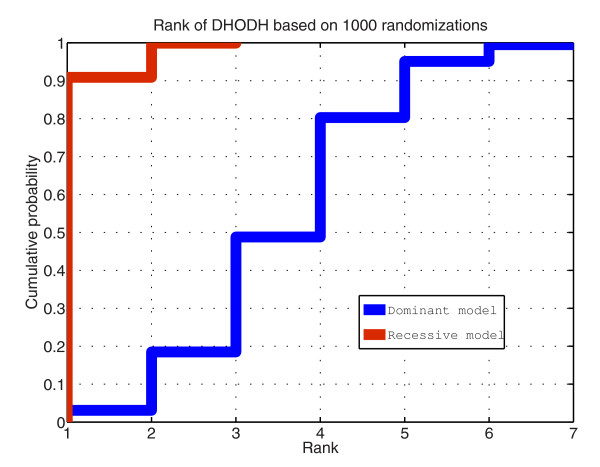
**Randomization scheme used for the semi-synthetic Miller data set**. We started from eight non-related previously acquired exome sequences and shuffled the sample identifiers linking a variant to a sample. By doing this the total number of variants and the number of variants per consequence was kept constant but the constitution of each of the exomes changed continuously. We then assigned four exomes to both control and case groups. In the case group we added two of the reported causal variants to each of the case samples. We then analyzed each of the randomizations with Annotate-it and looked at the resulting rank of *DHODH*. By repeating this randomization cycle 1,000-fold we calculated rank statistics.

We generated synthetic data sets of Miller syndrome by taking the following steps. We selected eight unrelated previously acquired exomes sequenced by Illumina GA2 and Illumina HiSEQ 2000. We aligned these exomes with BWA and called variants using the Genome Analysis Toolkit pipeline, both at default settings (Table 2). The sample identifiers of the variants were then randomly shuffled, maintaining the total number of variants, the number of variants per individual exome and the number of variants of each consequence type in the randomized data set (Figure 2). The eight synthetic exomes were then randomly assigned to either case or control groups so that each group contained four samples. Mutations found in the four published Miller syndrome cases were then added to each of the respective synthetic case samples. This synthetic case-control data set was then analyzed with Annotate-it using the following filter settings: minimum 10× coverage, minimum variant allele frequency of 20%, only 'novel' variants, variants not found in the 1000 Genomes Pilot project, and variants not occurring in the control samples. Only nonsynonymous, nonsense or essential splice site variants were considered. Ranking of the candidate genes was done based on the number of case samples in which the gene was considered a hit and the Phylop conservation score based on the multiple alignment of placental mammals. We calculated rank statistics based on 1,000 randomizations following the previous steps.

**Table 2:** Statistics of initial exomes used for randomizations

	Sample identifier							
	
Sample identifier	1	2	3	4	5	6	7	8
Platform	GAII	GAII	GAII	GAII	HiSEQ	HiSEQ	HiSEQ	HiSEQ
Essential splice site mutations	178	120	137	134	47	45	49	44
Nonsense mutations	99	88	95	108	101	60	68	68
Nonsynonymous mutations	8,864	7,468	8,039	8,246	7,925	6,715	6,487	6,560
Synonymous mutations	8,409	7,214	7,756	8,065	8,500	7,676	7,347	7,597
UTR mutations	3,839	3,216	3,080	3,264	4,264	1,443	1,421	1,367

Eight previously acquired exomes were used to generate 8,000 synthetic exomes by randomization. The initial exomes were sequenced by either Illumina GA2 or Illumina HiSeq 2000, then aligned with BWA and variants were called using the GATK pipeline.

## Results

We validated the use of Annotate-it using three independent datasets: 1) a semi-synthetic Miller syndrome dataset, 2) a Schinzel-Giedion syndrome dataset and 3) a Nicolaides Baraitser syndrome dataset. In Miller syndrome as in Schinzel Giedion syndrome the causative gene was previously discovered and thus served to see if results could be reproduced using Annotate-it. Due to the synthetic nature of the Miller syndrome set we were able to statistically estimate the efficiency of commonly used filtering approaches in the discovery of Mendelian disease genes. In the Nicolaides Baraitser syndrome dataset the causative gene was previously unknown to us and illustrates how additional phenotype-specific information can aid in the discovery of causative genes.

### Case study 1: semi-synthetic Miller syndrome data

To evaluate the impact of random neutral variation on finding the causative gene, we generated a synthetic data set modeled on published cases of Miller syndrome [5], a rare recessive disease caused by mutations in *DHODH*.

**Figure 3:**
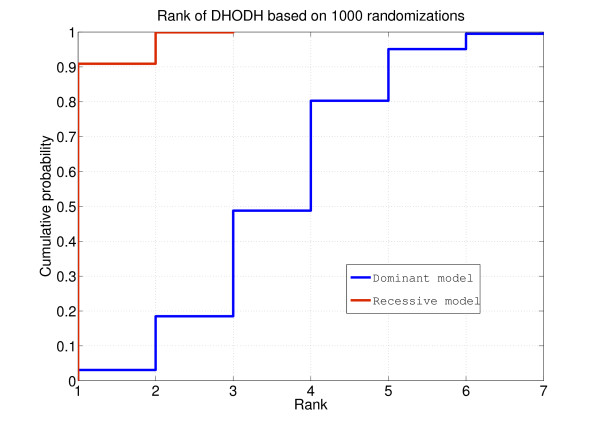
**Rank statistics for DHODH based on 1,000 randomizations**. The x-axis indicates the rank of *DHODH*, the y-axis indicates the cumulative probability of observing a rank lower than that marked on the x-axis.(red) Under the recessive model *DHODH *ranked in the top two candidate genes in 95% of the randomizations. (blue). Under the dominant model *DHODH *ranked in the top seven in 95% of the randomizations.

We considered two possible genetic models, which have been implemented in Annotate-it, when computing the rank of *DHODH*: the recessive model, meaning that a gene was only considered a hit in a sample if two different variants were found (assuming that a causal homozygous non-reference variant was highly unlikely), and the dominant model. Under the recessive model an average of 16 (± 2) candidate genes with hits in the 4 patients (41 (± 4), 105 (± 6),417 (± 12) candidate genes with hits in at least 3, 2, or 1 patient, respectively) were found. Out of these candidate genes, *DHODH *ranked in the top two in 95% of the randomizations (Figure 3). Under the dominant model an average of 113 (± 7) candidate genes with hits in the 4 patients (408 (± 12), 1132 (± 15),3417 (± 15) candidate genes with hits in at least 3, 2, or 1 patient, respectively) were found. Out of these candidate genes, *DHODH *ranked in the top seven in 95% of the randomizations. These data show that, despite random neutral variation, simple filtering and sorting criteria can dramatically decrease the number of genes to be validated in the case of Mendelian disorders.

### Case study 2: Schinzel-Giedion syndrome

In a study by Hoischen *et al. *[55] exome sequencing was used to identify causative mutations in Schinzel-Giedion syndrome. Because of the rarity of the disorder, the absence of gender bias, its mostly sporadic occurrence and the absence of cytogenetic imbalances, the disorder was believed to be caused by variants in a single or perhaps a few genes resulting in an autosomal dominant inheritance pattern. Exome sequencing of four patients, and validation in an additional four patients by Sanger sequencing, has identified *de novo *mutations in *SETBP1 *to be causative for the disease. These mutations were clustered in a highly conserved region of the gene and are found in an 11-bp stretch. In the previous study, mutations were filtered based on dbSNP build 130 for novel variants and only nonsynonymous (mutations changing the amino acid composition of the protein), nonsense (mutations resulting in either a gain or loss of a stop codon) or essential splice site variants (mutations located within two bases of the intron/exon boundary) were considered. Previously generated in-house data were used to filter out variants found in samples not related to the phenotype. This was done under the assumption that because of the rarity of the disorder, it was unlikely that these control samples would contain the same causal variant.

We reanalyzed the data using Annotate-it and applied default filters to validate the effectiveness of our methodology. We uploaded the variant files for annotation on the hg18/NCBI36 build of Annotate-it and filtered variants using two different settings: a cross-sample comparison at the gene level and a comparison at the exon level. The only major difference between both strategies is that instead of looking for candidate genes hit by variants across the patients, in the second approach we also look for specific exon hits across patients. For both strategies, we filtered out variants present in dbSNP (build 130) and the 1000 Genomes Project pilot studies (July 2010 release). We only selected variants with nonsense, nonsynonymous, and essential splice site consequences. We did not set any further filters, so as to recreate the scenario in which no further information was available to the researcher. Even though stricter filter settings would reduce the size of the resulting gene lists, we decided that manual inspection of all available information would allow us to select potential candidate genes without filtering *a priori *and potentially suffering false negative results that could lead to missing the implicated gene.

Both filtering approaches resulted in either a list of 11 candidate genes hit across the 4 patients (or 30, 91, 793 candidate genes in, respectively, at least 3, 2, or 1 patient) or 13 candidate exons across the 4 patients (or 38, 81, 840 candidate exons in, respectively, at least 3, 2, or 1 patient). Sorting the gene list on number of samples implicated and maximum Phylop scores for placental mammals in the found variants, *SETBP1 *ranked at the top of the list as the prime candidate (Table 3). Further inspection of *SETBP1 *revealed the cluster of variants in a highly conserved region in the graphical view (Additional file 4), which might be indicative of gain-of-function mutations. Other genes that were hit across the four samples were also found but were deemed less likely because of either being hypervariable (for example, *CDC27*) or having variants in regions of lower conservation. In the exon-based approach one of the *SETBP1 *exons was ranked in the top ten exons hit across the four patients. Other high ranking exons were deemed unlikely candidates because of the high number of variants found and were likely hypervariable regions or pseudogenes. This approach is theoretically more sensitive to distinguish causative exons, as neutral variation is less likely to be clustered in individual exons across samples, even though it remains susceptible to the previously mentioned regions of increased variability.

**Table 3:** Top ten candidate genes in the Schinzel-Giedion case study

Rank	Gene	Number of samples	Number of unique variants	Total number of variants	Haploinsufficiency prediction	Maximum Phylop (placental mammals)
1	*SETBP1 *	4	2	3	0.721	3.455
2	*CDC27 *	4	5	13	-	3.106
3	*CTBP2 *	4	3	8	-	2.805
4	*PRB1 *	4	2	5	-	1.458
5	*KIR2DL1 *	4	1	2	-	1.05
6	*FLG *	4	5	6	-	1.034
7	*OR11H1 *	4	0	1	-	0.856
8	*KIR2DL3 *	4	4	6	-	-0.326
9	*CDCP2 *	4	0	1	-	-0.365
10	*NBPF12 *	4	0	2	-	-0.411

The data were analyzed using Annotate-it. Genes were considered a hit if they contained either nonsynonymous, nonsense or essential splice site variants that were not present in dbSNP or found in the 1000 Genomes Project. Genes were then sorted based on number of affected samples in which the gene was a candidate and conservation in placental mammals. Additionally, the total number of variants present across all samples and the number of variants unique to only one sample is given.

### Case study 3: Nicolaides-Baraitser syndrome

Additionally we used Annotate-it to unravel the etiology of the previously unsolved Nicolaides-Baraitser syndrome (NBS) [56]. NBS is characterized by intellectual disability, sparse hair, distinctive facial characteristics, and distal-limb anomalies. The extremely rare sporadic recurrence of the disease and the lack of bias towards any gender suggested the cause of the disease to likely be autosomal dominant *de novo *mutations. We collected DNA from four unrelated cases and performed whole-exome sequencing. We ran the analysis in Annotate-it using default filters and parameters (nonsense, splice site, and nonsynonymous mutations not present in any of the available population databases). This resulted in a list of 296 candidate genes sorted based on the number of samples having mutations in the given gene and the number of mutations pertaining to a single sample for that gene. The top ranked gene, *ACVR2A*, was the only candidate gene with variants present in the four samples containing the same variant. Recurring mutations are more likely to be population-specific polymorphisms, and thus not captured by aspecific population databases, than *de novo *variation, which is believed to randomly occur throughout the genome and therefore has a very low probability of mutating the same position four times in non-consanguineous individuals. The second best ranking gene, *SMARCA2*, contained unique heterozygous nonsynonymous mutations in three of the four samples. Further investigation of associated gene ontologies and associated phenotypes computed by AGeneApart revealed this gene to be involved in chromatin remodeling and facial abnormalities, making it an interesting candidate for validation. Sanger sequencing of the samples and their respective parents revealed the three variants to be *de novo *events. Additional resequencing of *SMARCA2 *in 44 individuals revealed heterozygous mutations in the same carboxy-terminal helicase domain in 36 patients, hereby validating *SMARCA2 *as the causative gene for Nicolaides-Baraitser syndrome. Later published studies discovered the etiology of the phenotypically related Coffin-Siris syndrome to be caused by similar mutations in *SMARCB1 *and *ARID1B*, which are direct interaction partners of *SMARCA2 *in the SWI/SNF chromatin remodeling complex [57,58]. Based on these findings we added functionality to Annotate-it to find causative mutations in protein complexes rather than in a strictly monogenic setting. By doing this we discovered that one of eight patients diagnosed with NBS for which we performed whole-exome sequencing carried a mutation in a gene identified in Coffin-Siris syndrome. Although the phenotype of NBS is strongly overlapping with that of Coffin-Siris syndrome, the latter differs by the presence of hypoplastic or absent fifth finger nails with or without hypoplasia of the terminal phalanges, the absence of swelling of the finger joints, short metacarpals and broad terminal phalanges and internal organ malformations are more common. Yet due to the large overlap of features, both syndromes are hard to classify [59], especially at a young age, which could explain the misclassification of this particular sample.

## Discussion

### Context

A multitude of publicly available computational tools exist in order to assist bioinformaticians and geneticists by facilitating the analysis of single nucleotide variants at the level of annotation, interpretation or both. Yet most tools focus only on particular aspects of the analysis pipeline (Table 1) and are thus only usable under certain experimental circumstances. Furthermore, all described tools suffer from several drawbacks, such as 'red queen' annotation problems, cross-sample analysis, phenotype-naive interpretation and general data management problems, such as data compatibility and data sharing [60].

### Annotate-it

In this paper, we describe Annotate-it, a versatile framework for the analysis of multisample single nucleotide substitution data generated by next-generation sequencing. Annotation of samples is performed on the server side, eliminating the need for the installation of complex tools and annotation sources by the end-user and automatically keeping those annotations up to date.

The query and filtering interface enables the geneticist to quickly test different genetic hypotheses (recessive, dominant, *de novo*) across multiple samples and aggregates available information at the gene and variant level, facilitating the manual revision of candidate gene lists. We also provide a suite of more complex analysis techniques, such as aggregate functionality scores, phenotype-specific gene prioritization, and statistical methods for disease-gene finding in case/control studies without any need for data reformatting or computational expertise. By merging different state-of-the-art analysis techniques we aim to deliver a Swiss-knife type tool that can be used in many different experimental contexts. By unifying the interface and centralizing the data the user is able to perform multifaceted analyses without any need for additional data management or formatting and without requiring any computational expertise.

### Case studies

To validate our approach we simulated an experiment with randomized exomes and published variants found in Miller syndrome patients. We computed ranking statistics showing that the causative gene ranks highly, even with a large amount of random neutral variation. This happens even when considering a relatively small set of patient exomes. In a second benchmark, we reanalyzed a published case study of the Schinzel-Giedion syndrome and identified the causative gene, *SETBP1*, as the top-ranked gene, using default parameters. Further inspection reinforced the previous finding that novel nonsynonymous variants across patients are clustered in a small 11-bp stretch. We applied the same approach in a previously unpublished study of NBS and found *SMARCA2 *as one of the top-ranking genes. This gene was then further validated to be the causative gene in this disease. Furthermore, we show in this case that gene prioritization methods can aid in the prioritization of mutated candidate genes by linking these genes to their associated phenotypes. Additionally, we show that in phenotypically overlapping disorders, leveraging protein-protein interaction data could prove useful in deciphering neighborhoods of mutated genes.

### Future perspectives

Although the framework as presented here is fully able to support the discovery of causal genes of rare genetic disorders, our approach would benefit further from several critical features that we will investigate in future work. First, the confidentiality of human clinical data is a concern of many clinical geneticists and is still a topic of debate in most research centers. We aim to develop future releases of Annotate-it using a client-server architecture, so that sample and genotype information is dealt with on a locally installed client that automatically synchronizes genomic (but not clinical) data (that is, single nucleotide variant chromosome, position, and variant allele) with a server that holds the most up-to-date annotation information. A public client will be available for sharing data across research centers. Secondly, we designed Annotate-it with protein coding single-nucleotide substitutions in mind as proof-of-concept. We aim to further expand the scope of the methodology to encompass annotation of non-coding regions and other types of variation, such as insertions and deletions of varying lengths.

## Conclusions

In this paper we describe the application of Annotate-it to study rare monogenic Mendelian disorders caused by rare, highly penetrant variants in the population. Because sequencing (as opposed to classical array-based genome-wide association studies) detects rare (less than 0.01% of the general population), infrequent, and frequent variants (over 1%), extensions of our strategy will be useful in resolving oligogenic diseases caused by a combination of infrequent variants of intermediate penetrance. Discovering the disease genes in such challenging cases will require good models of (1) the underlying population genetics (to avoid confounding effects from population structure), (2) the functional impact of variants on protein function and on regulation (to weed out passenger mutations), and (3) the biological pathways involved (to detect causative variants through their effect on pathways rather than individual genes). This is a primary research goal for the near future.

Current approaches to identifying causal variants by patient sequencing, including ours, can reliably interpret only the most straightforward subset of the variome (nonsense, missense, and splice-site variants). For example, synonymous variants, variants in regulatory, intronic, and intergenic regions, and variants in microRNAs remain highly challenging. Future experimental and *in silico *studies assessing the impact of such variants will allow us to develop computational strategies to bridge the gap in data interpretation between the exome and the complete genome. In particular, refinements in the mapping of regulatory regions and other functional elements through functional studies, evolutionary conservation, and *in silico *models will be essential to enable variant interpretation beyond the coding exome. Furthermore, the integration of large-scale structural variation will be crucial in a comprehensive study of the variome. Such integration will be challenging because structural variation inherently requires different types of data structure, visualization, and analysis methods. A high-level meta-analysis of variation at multiple scales should provide researchers with a complete picture of individual genomes. Making such complex, integrative analyses effective is a major challenge for the computational biology community.

Given the positive reactions from initial users, we believe that Annotate-it can play a significant role in the prioritization and identification of candidate genes in Mendelian disease. In the future, we aim at broadening its scope by increasing the annotation information available, the effectiveness and scope of our filtering analysis methods, and the robustness and ease of use of our web interface.

## Availability

Annotate-it is freely available at [61]. Bug reports can be made and feedback accessed at [62].

## Requirements

Annotate-it has been tested on the following browsers: Google Chrome (build 11.0.696.71 or later), Mozilla Firefox (version 3.6 or later), Safari (version 5.0 or later). Microsoft Internet Explorer is currently not supported.

## Abbreviations

bp: base pair; NBS: Nicolaides-Baraitser syndrome; OMIM: Online Mendelian Inheritance in Man; SNP: single nucleotide polymorphism.

## Competing interests

The authors declare that they have no competing interests.

## Authors' contributions

AS developed and maintained the system, prepared the first draft of the manuscript and reanalyzed the case study data; AS, JKJVH, YL, BN, LCT, RS, KD, JRV, YM and JA contributed conceptually to the project. LCT precomputed all gene prioritizations; AS, RS, GAP and JA contributed to the visual interactive interface; AS, JKJVH and BN tested the software. JRV and KD supervised the clinical genetic aspects of the project. YM and JA were the main supervisors of the project. All authors contributed equally to the final manuscript.

## Supplementary Material

Additional file 1 **Screenshot of *Annotate-it*'s filter settings pane**. Through the web interface the user can easily analyze imported data sets through a simple point and click interface. Samples can be easily added or excluded from the analysis. Filter settings such as minimum coverage, presence in dbSNP and 1000 Genomes Project and uniqueness can be applied and different sorting schemes can be devised and then applied on the selected samples. Click here for file

Additional file 2 **Screenshot of Annotate-it's gene list view**. After selecting samples to be analyzed and filtering and sorting criteria a resulting gene list is outputted. This gene list contains information on the amount of selected samples containing unfiltered variants and conservation scores. A tab with distribution plots is also provided (not shown here). Genes in this list are clickable and doing so returns more gene- and variant-specific information for that respective gene (Additional file 3). Click here for file

Additional file 3 **Screenshot of Annotate-it's gene details view**. Mutated genes can be further inspected to show additional information such as gene ontology, publications, associated pathways, associated phenotypes through gene prioritization, interaction partners (and their mutations) and involved protein complexes (and their mutations). Also detailed information on the mutations contained in that gene is given. Click here for file

Additional file 4 **Screenshot of Annotate-it's gene view visualization**. This visualization shows the clustering of variants in an 11-bp stretch of a single highly conserved exon of *SETBP1 *between position 40783844 and 40787303. Conservation scores are given for the complete exon on the primate, mammal and vertebrate level. Nonsynonymous mutation are marked as light blue circles each belonging to one of the four patients (with sample identifiers 40816, 43664, 48062, 54126). Click here for file
