# Supplementary figures and images for: Annotate-it: a Swiss-knife approach to annotation, analysis and interpretation of single nucleotide variation in human disease
Source: Genome Med. 2012 Sep 26;4(9):73. doi: 10.1186/gm374 (PMC3580443; doi:10.1186/gm374)

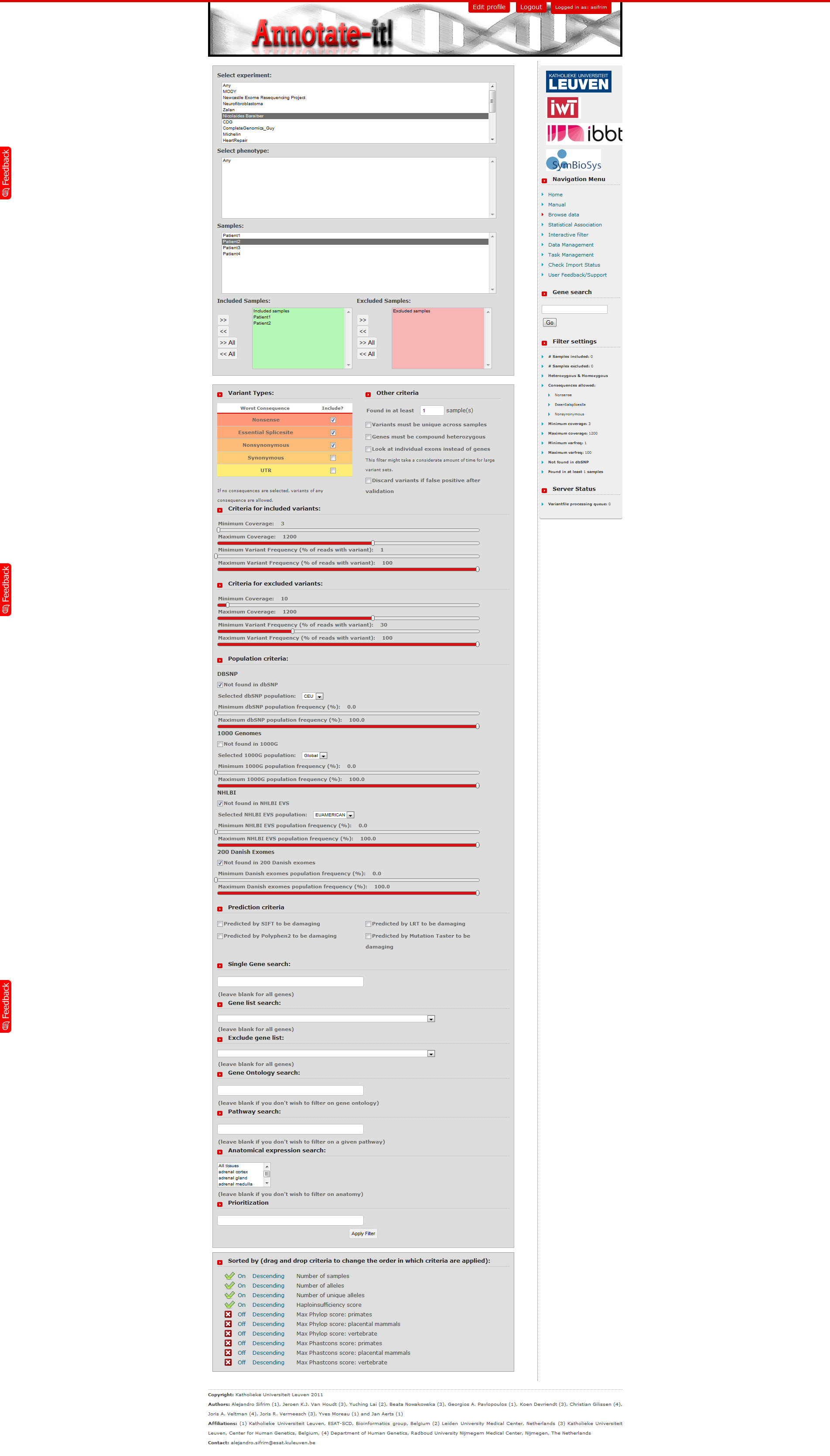

Supplement: Additional file 1 — Screenshot of Annotate-it's filter settings pane. Through the web interface the user can easily analyze imported data sets through a simple point and click interface. Samples can be easily added or excluded from the analysis. Filter settings such as minimum coverage, presence in dbSNP and 1000 Genomes Project and uniqueness can be applied and different sorting schemes can be devised and then applied on the selected samples. [file gm374-S1.PNG]

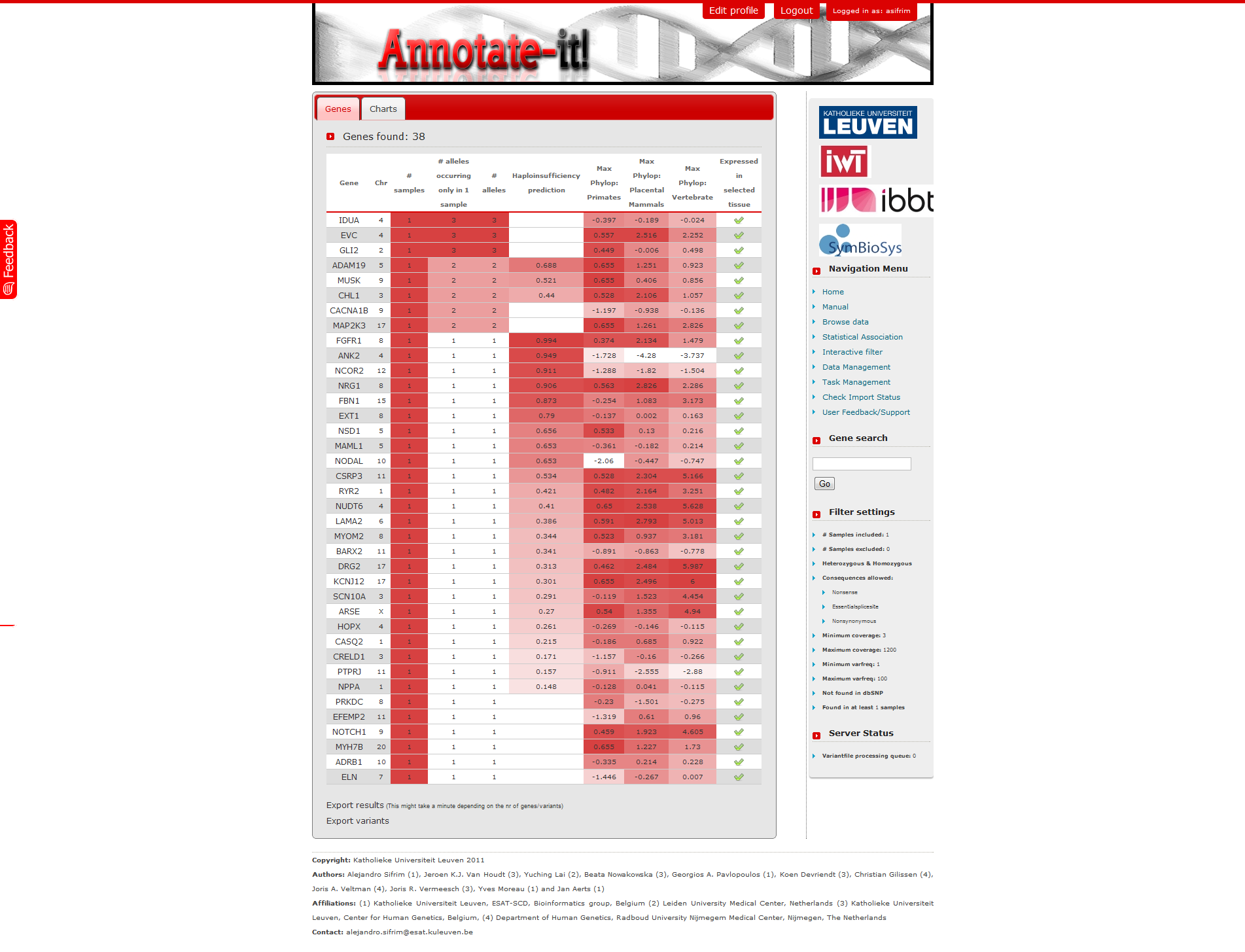

Supplement: Additional file 2 — Screenshot of Annotate-it's gene list view. After selecting samples to be analyzed and filtering and sorting criteria a resulting gene list is outputted. This gene list contains information on the amount of selected samples containing unfiltered variants and conservation scores. A tab with distribution plots is also provided (not shown here). Genes in this list are clickable and doing so returns more gene- and variant-specific information for that respective gene (Additional file 3). [file gm374-S2.PNG]

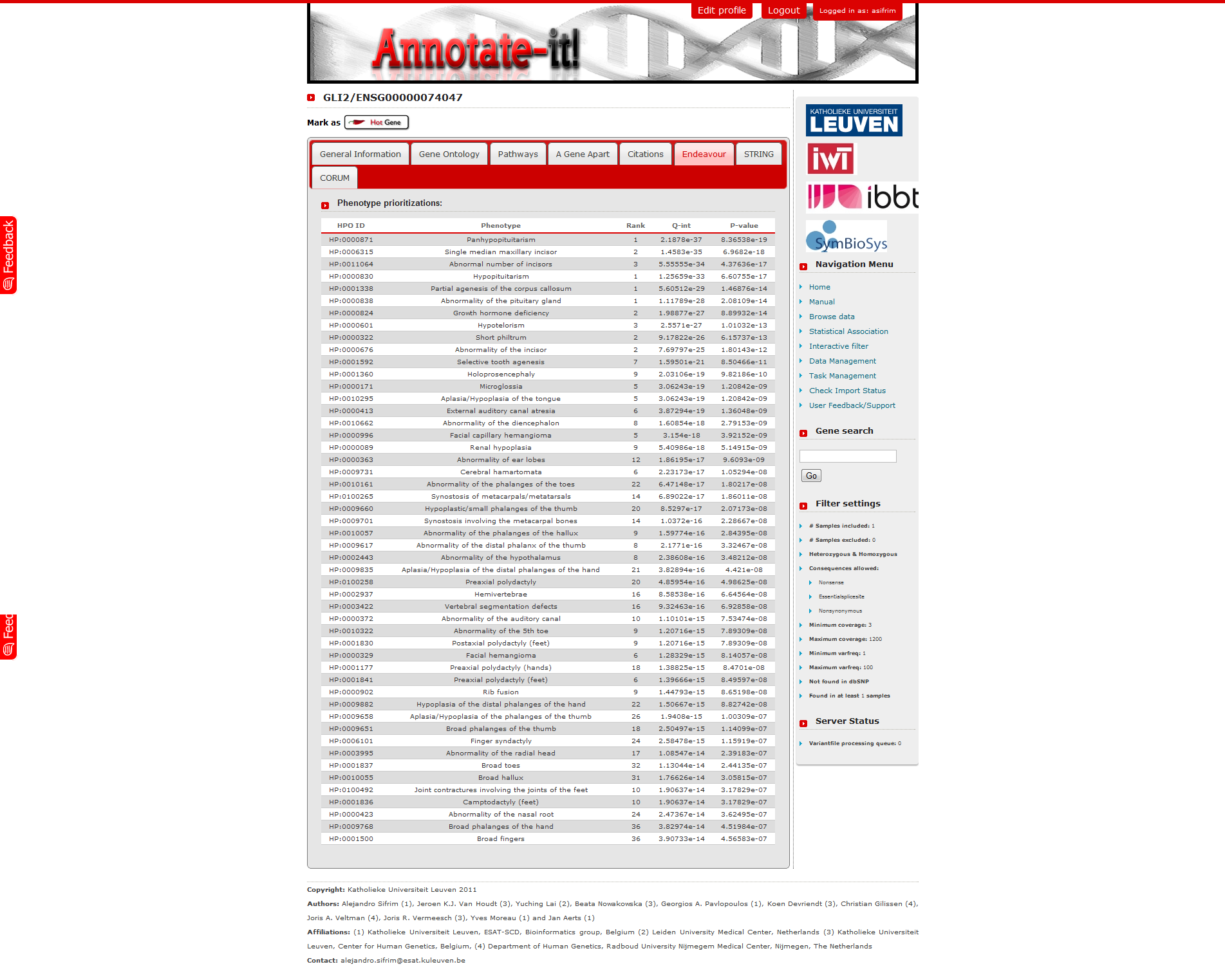

Supplement: Additional file 3 — Screenshot of Annotate-it's gene details view. Mutated genes can be further inspected to show additional information such as gene ontology, publications, associated pathways, associated phenotypes through gene prioritization, interaction partners (and their mutations) and involved protein complexes (and their mutations). Also detailed information on the mutations contained in that gene is given. [file gm374-S3.PNG]

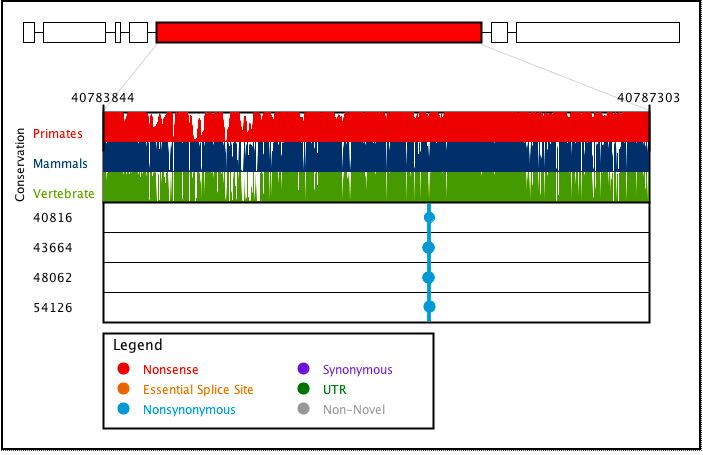

Supplement: Additional file 4 — Screenshot of Annotate-it's gene view visualization. This visualization shows the clustering of variants in an 11-bp stretch of a single highly conserved exon of SETBP1 between position 40783844 and 40787303. Conservation scores are given for the complete exon on the primate, mammal and vertebrate level. Nonsynonymous mutation are marked as light blue circles each belonging to one of the four patients (with sample identifiers 40816, 43664, 48062, 54126). [file gm374-S4.PNG]
